# Physical exercise, gut, gut microbiota, and atherosclerotic cardiovascular diseases

**DOI:** 10.1186/s12944-017-0653-9

**Published:** 2018-01-22

**Authors:** Jingyuan Chen, Yuan Guo, Yajun Gui, Danyan Xu

**Affiliations:** Department of Cardiovascular Medicine, The Second Xiangya Hospital, Central South University, Changsha, Hunan 410011 China

**Keywords:** Atherosclerosis, Physical exericise, Gut microbiota

## Abstract

Arteriosclerotic cardiovascular diseases (ASCVDs) are the leading cause of morbidity and mortality worldwide and its risk can be independently decreased by regular physical activity. Recently, ASCVD and its risk factors were found to be impacted by the gut microbiota through its diversity, distribution and metabolites. Meanwhile, several experiments demonstrated the relationship between physical exercise and diversity, distribution, metabolite of the gut microbiota as well as its functions on the lipid metabolism and chronic systematic inflammation. In this review, we summarize the current knowledge on the effects of physical exercise on ASCVD through modulation of the gut microbiota and intestinal function.

## Background

Cardiovascular diseases (CVDs) are the leading cause of morbidity and mortality worldwide [1]. Arteriosclerotic CVD (ASCVD) manifests as chronic ischemia in the affected organs over time or as acute symptoms such as myocardial infarction and ischemic stroke as a consequence of plaque destabilization or thrombus formation [2, 3].

Physical exercises could independently decrease the risk of ASCVD and also has a positive intensity-related impact on other cardiovascular risk factors, such as hyperlipidemia, hypertension, abdominal obesity, diabetes, and psychosocial factors. In addition, there is a positive correlation between exercise intensity and protective effects against ASCVD. Most authorities recommend higher-intensity aerobic exercise and resistance training to prevent and treat CVDs [4, 5].

Although physical exercise had been commended by lots of guidelines and expert consensus for its prevention and protection effects on ASCVD, its underline mechanisms were still not well understood. Several beneficial effects that acted on ASCVD had been found. First, the increased flow-mediated shear stress on the artery walls during exercise improves endothelial function. Second, aerobic exercise training is associated with reduced serum C-reactive protein levels. Furthermore, endurance exercise decreases blood pressure and serum triglyceride (TG) levels and improves high-density lipoprotein cholesterol levels (HDL-C), insulin sensitivity, and glucose homeostasis. Endurance exercise training also has potential anti-ischemic effects and increases coronary flow by augmenting capillary density and blood flow area [6].

Recently, ASCVD were found to be impacted by the gut microbiota through its diversity, distribution and metabolites. There were also some relations between gut microbiota, gut inflammation and ASCVD. Meanwhile, several experiments demonstrated the relationship between physical exercise and gut microbiota, and its influences on ASCVD risk factors such as the lipid metabolism and chronic systematic inflammation. In this review, we summarize the current knowledge on the effects of physical exercise on ASCVD through modulation of the gut microbiota and intestinal function.

## Gut microbiota and ASCVD

### Species, distribution and diversity of gut microbiota and ASCVD

Gut microbiota is the general term for bacterial microorganisms in the human digestive tract. The variety of microbes colonizing the human gut is almost 10 times that of the total cells in a human, and the genetic materials are more than 150-fold that of humans [7]. Classified by phyla, the gut microbiota mainly comprises *Firmicutes* and *Bacteroides*, which account for 80%–90% of all gut microbes, followed by *Actinobacteria* and *Proteobacteria*. Among these phyla, *Firmicutes* mainly includes *Ruminococcus*, *Clostridium*, *Lactobacillus*, *Eubacterium*, *Faecalibacterium*, and *Roseburia*, while *Bacteroides* mainly comprises *Prevotella* and *Xylanibacter* [8].

Researchers who screened the human gut microbiota composition based on the difference in bacteria gene discrepancy according to microorganism metagenome found that there are three types of human gut microbiota rather than a simple random assortment [9, 10]. Accordingly, a new typing method was developed that stratifies gut microbiota into three enterotypes as determined by the abundance of key bacterial genera, namely, type 1 (mainly *Bacteroides*), type 2 (mainly *Prevotella*), and type 3 (mainly *Ruminococcus*, and smaller proportions of *Bacteroides*, *Roseburia*, and *Blautia*). Moreover, enterotypes are not related with age, sex, weight, or race, but with eating habits and means of energy utilization and vitamin synthesis. Relevant research on atherosclerosis and gut microbes has shown that patients with atherosclerosis are mainly enterotype 3, with a minority of enterotype 1 [10].

In the following studies they found the atherosclerotic plaque area in female rats was positively correlated with *Clostridiales* (*Firmicutes*), *Ruminococcus* (*Firmicutes*,), and *Lachnospiraceae* (*Firmicutes*), and was negatively correlated with the S24–7 family of *Bacterioidetes*, which is similar to human enterotype research findings [11].

Intensive study of bacterial genera and families has proven that some rare genera or families are associated with ASCVD. A method based on the gut metagenome showed that the proportion of the genus *Collinsella* is increased in sympathetic atherosclerotic patients, while the proportions of *Roseburia* and *Eubacterium*are greater in healthy individuals [10].

### Metabolites of gut microbiota and ASCVD

#### Short chain fatty acid (SCFA) and ASCVD

Short-chain fatty acids (SCFAs) are the major end products from the microbial degradation of carbohydrates and protein in the gut. The majority of SCFAs are absorbed from the gut and metabolized in various body tissues, contributing to some important physiological processes, especially effects daily energy requirements [12, 13].

Through the absorption and metabolism of SCFA, the host is able to obtain energy from foodstuffs that are not fully digested. SCFAs have numerous effects throughout the body, such as affecting epithelial cell transport and metabolism, growth and differentiation, and controlling lipid and carbohydrates metabolite in hepatocytes and providing energy sources [14]. For instance, propionates could reduce the cytokine-induced expression of cytokine induced adhesion molecules such as vascular cell adhesion molecule 1 (VCAM-1) and intercellular adhesion molecule 1 (ICAM-1) in endothelial cells by inhibiting nuclear factor-κ B (NF-κB) [15, 16], lowers blood glucose and cholesterol, alter lipid metabolism [17], inhibit cholesterol synthesis in hepatocytes [18], which are all pathogenesis and risk factors of ASCVD.

#### Trimethylamine N-oxide (TMAO) and ASCVD

Other than the abovementioned effects, the gut microbiota participate in the pathology of ASCVD through the metabolic product trimethylamine (TMA). In the setting of specific dietary nutrients characterized by TMA (e.g., choline, phosphatidylcholine, carnitine), the gut microbiota participate in the formation of the proatherogenic compound TMAO.

An accumulating amount of evidence suggests that, in humans, elevated concentrations of plasma TMAO are a marker of increased cardiovascular risk. Tang et al. [19] enrolled 4007 adults undergoing elective diagnostic cardiac catheterization to determine their fasting serum TMAO levels. Participants who had major adverse cardiovascular events had higher baseline levels of TMAO [20]. Compared with participants in the lowest quartile, those in the highest quartile had significantly increased risk of adverse cardiovascular events, indicating that TMAO remained a significant predictor of cardiovascular events risk [21].

Wang et al. first found that TMAO potentially promotes atherosclerosis by enhancing foam cell formation and decreasing reverse cholesterol transport (RCT) [22]. Subsequently, they screened a structural analog of choline, 3,3-dimethyl-1-butanol (DMB), which non-lethally inhibited TMA formation and decreased foam cell and atherosclerosis [11].

A detailed mechanism in vitro confirmed the mechanistic role of TMAO on foam cell formation through the upregulation of scavenger receptor A1 (SR-A1), ATP-binding cassette transporter A1 (ABCA1) and CD36 [22, 23]. However, at high baseline TMAO levels (> 0.05 ppm), the decrease in TMAO levels is associated with reduced aortic lesion area. Furthermore, TMAO and aortic lesion area in apolipoprotein E–null (apoE−/−) mice expressing human cholesteryl ester transfer protein (CETP) were not related. These results demonstrate that TMAO does not affect foam cell formation or endothelial cell dysfunction, the two first steps in atherosclerotic disease progression [24]. The discrepancies between these studies may be attributed to differences in diet composition, intervention drug dosage, and the sex and species of the experimental mice.

In patients with ASCVD, increased TMAO levels are associated with myocardial infarction, stroke, and all-cause mortality, mainly because TMAO enhances platelet aggregation and thrombosis. The most recent research shows that TMAO promotes platelet hyper-responsiveness by enhancing the inositol-1,4,5-trisphosphate (IP3) signaling pathways and activating calcium ion (Ca2+) release from intracellular Ca2+ stores [25].

### Gut microbiota and risk factors of ASCVD

#### Gut microbiota and lipid metabolism

*Bacteroidetes* is more common and *Firmicutes* is less ubiquitous in obese people than in lean people [26]. In addition, germ-free mice have lower fatty acid oxidization and decreased lipolysis [27, 28]. Metabolomic research that compared the blood lipid profiles between germ-free mice and typical breeding mice confirmed this conclusion, finding lower serum TG levels in germ-free mice, which was in accordance with the increased TG clearance [29].

Alterations in the intestinal microbiota, especially lactic acid bacteria (LAB), have yielded important modifications to lipid metabolism in animals [30–32] and humans [33]. Some yeast species can remove cholesterol [34], besides, *Bifidobacteria* decreases cholesterol levels by assimilation and precipitation [35], and is associated with serum HDL-C levels [36]. Furthermore, many experiments have shown a relationship between *Erysipelotrichi* and host lipid metabolism [36, 37]. In addition, gut microbial modification by antibiotic or probiotics use ameliorates dyslipidemia [38].

Research in vitro and in vivo has clarified the detailed mechanisms of the cholesterol-lowering effects of the gut microbiota, such as decreased gut assimilation, inhibition of the connection between cholesterol and the cell surface [39, 40], influencing the production of short-chain fatty acids [41], and accelerated bile acid and catalytic enzyme deconjugation [42, 43].

#### Gut microbiota, endotoxins, and chronic systematic inflammation

Gram-negative bacteria colonization leads to abundant endotoxins, especially LPS, in human and animal gut lumina. Several studies have reported low levels of serum LPS in humans, demonstrating that LPS is absorbed at a slow rate from the gastrointestinal lumen [44]. One major means of LPS absorption is infiltration of the tight junctions of the intestinal epithelium.

Decreasing tight junction protein levels augments LPS permeation, causing chronic inflammation such as visceral fat inflammation and macrophage infiltration [44, 45], which are risk factors of ASCVD [46].

Endotoxin levels are increased in obese rats and are accompanied by decreased intestinal endothelial zonula occludens-1 (ZO-1) and occludin levels [47]. The levels of serum endotoxins (especially LPS), proinflammatory cytokines, and hepatic inflammation in mice fed a HFD were decreased by broad-spectrum antibiotics, which altered the gut microbiota. These effects are associated with increased tight junction protein levels and decreased intestinal permeability [45]. Moreover, probiotics such as *Lactobacillus* improved the expression of tight junction proteins and ameliorated the absorption of LPS and its proinflammatory effects by improving the gut innate immune response [48]. Human *Prevotella histicola* reduces intestinal permeability and inflammation by upregulating the expression of enzymes that produce antimicrobial peptides and the expression of the tight junction proteins ZO-1 and occludin [49].

Among different inflammatory pathways, innate immunity, and particularly toll-like receptor (TLR)-activated pathways, have played an important role in the pathological process. TLR2 and TLR4 are the main receptors of LPS [50, 51], and TLR5 is a specific receptor of flagellin [52]. Gene mutation and gene knockout in TLR2 or TLR4 decrease TLR4 signaling and ameliorate atherosclerosis. Twelve-week treatment with broad-spectrum antibiotics reduces intestinal microbiota diversity and inhibits TLR4 signaling [53]. TLR2 and TLR4 participate in tight junction protein regulation [44, 53, 54]. Gut microbiota dysbiosis also increases the levels of endotoxin and flagellin released by gram-negative bacteria, combining with TLR5 and contributing to intestinal endothelial inflammation and injury. Moreover, this disruption of the intestinal environment is modestly related with damage to the tight junction proteins claudins (CLDN) and occludins [55].

## Physical exercise and gut microbiota

The modern lifestyle, such as diet and exercise, influences gut microbiota composition and the health of the host to an extent. Compared with research on diet, research on the relationship between physical exercise and gut microbiota is less well developed.

### Physical exercise, microbial distribution, and diversity

There are several studies demonstrate that physical exercise increases microbiota diversity and modulates its distribution (Table 1). Bacterial diversity is decreased in sedentary elderly individuals as compared with elderly individuals who have physical exercise. The gut microbiota of professional rugby players were more diverse than that of non-athlete healthy subjects [56]. Santacruz [57] compared changes in the gut microbial distribution of obese adults who had moderate to severe aerobic exercise for 10 weeks and found increased *Bacteroidetes* and decreased *Firmicutes*. These studies suggest that exercise can alter microbiota diversity and distribution in humans.

**Table 1:** Studies about exercise and gut microbiota

Model	Exercise	Changes of microbial groups or SCFA	Position	Reference
diet-induced obesity C57 BL/6 mice	high-intensity interval training (HIIT) for 6 weeks	↑*Bacteroidetes/Firmicutes* ↑Fecal microbiota genetic capacity	distal gut feces	[59]
Normal diet SD rats	voluntary access to exercise (i.e., wheel running)	↑*Bacteroidetes* ↓*Firmicutes*↑*Lactobacillus, Bifodobacterim* ↑*Blautia Coccoides Eubacterium rectale* ↓*Clostridium Enterococcus*	feces	[70]
6w Male Wistar rats	voluntary running exercise	↑butyrate ↑*phylum of Firmicutes (SM7/11, T2-87)*	colon	[62]
Rugby players	rugby	↑microbial diversity ↑*Akkermansia* ↑*Firmicutes* in athletes ↓*Bacteroidetes*	feces	[71]
Diabetic Mice(db/db)	low-intensity treadmill running	↑ *Firmicutes* ↓ *Bacteroides/Prevotella*	cecal feces	[60]

Experiments in rats have also yielded the same result, in which there was an effect on microbial distribution between HFD and voluntary exercise. In that study, exercise increased the percentage of *Bacteroidetes* and decreased the percentage of *Firmicutes* regardless of diet; moreover, the ratio of *Bacteroidetes* to *Firmicutes* correlated inversely with the amount of exercise performed [58]. In a recent experiment, Emmanuel et al. [59] found that high-intensity interval training increased the *Bacteroidetes* to *Firmicutes* ratio of the distal gut and fecal microbiota during diet-induced obesity.

*Firmicutes* and *Bacteroidetes* are the most abundant microorganisms at phylum level and account for more than 90% of the total microbiota. Moreover, intestinal flora are complicated, spurring intensive research on particular populations but not on the *Firmicutes* to *Bacteroidetes* ratio.

Research on the effect of physical exercise on diabetic and normal mice has shown that physical exercise correlates with decreased proportions of *Bacteroides*/*Prevotella* spp. and *Methanobrevibacter* spp. and increased proportions of *Lactobacillus* spp. [48].

The most recent study comparing the effects of physical exercise on different diets showed that exercise altered the gut microbiota distribution in both HFD and normal diet, and the unique genera associated with physical exercise are *Faecalibacterium prausnitzii (Firmicutes), Clostridium spp. (Firmicutes),* and *Allobaculum spp. (Mycoplasmataceae)* [60]. Moreover, the proportions of *Allobaculum* spp. and *Clostridiales* were increased after exercise (either HFD or normal diet) as compared with sedentary groups (either HFD or normal diet), and *F. prausnitzii* was only present in the exercise groups regardless of animal species [61], which is consistent with previous study findings.

### Physical exercise and SCFA

There are also a few studies proved that exercises could promote formation of SCFA. In animal models, it has been observed that running exercise increases fecal butyrate levels, and this change is associated with changes in butyrate producer bacteria groups [62]. Voluntarily wheel exercise was also found to increase cecal acetate and propionate. Therefore, increased SCFAs production induced by microbiota profile changes could be one of the mechanisms by which physical exercise promotes health, since SCFA butyrate has the ability to inhibit histone deacetylases, and subsequently it has an impact on gene regulation, immune modulation, intestinal barrier regulation, oxidative stress reduction, diarrhea control, visceral sensitivity, and intestinal motility modulation [63], which all participate in the modulation of ASCVD. Moreover, SCFAs catabolited by the microbiota were found to activate AMPK pathway that controls the activity of various factors implicated in the regulation of cholesterol levels and metabolism of lipids and glucose in the muscle [64].

### Physical exercise influences ASCVD risk factors through intestinal functions

There are several evidences that habitual physical activity is anti-inflammatory and protective against developing chronic inflammatory diseases. Recent studies have related intestinal dysbiosis to pathogenic microbes and there are also results indicates increased inflammatory disease susceptibility [65]. There were some animal and human researches demonstrate that exercise may have a beneficial role in preventing and ameliorating chronic inflammatory diseases by having an effect on gut immune function and microbiome characteristics. These researches showed that different forms of exercise training differentially impact the severity of intestinal inflammation during an inflammatory circumstance and could be linked to gut immune cell homeostasis and microbiota-immune interactions.

Research on the field of physical exercise and intestinal permeability mostly shows that strenuous exercise increases gut permeability induced by intestinal ischemia. This is the cause of abdominal discomfort and diarrhea in athletes while they are competing [66, 67]. However, these findings may not be the only connection between exercise and gut permeability. A decrease of about only 50% in the intestinal blood stream causes intestinal permeability deterioration. Moreover, the intestinal blood stream of foxhounds did not decrease further after 8–12 weeks of exercise training [68]. These findings indicate that the increased gut permeability induced by intestinal ischemia is associated with the intensity, duration, and adaptability of exercise.

The mRNA levels of CLDN1 and ZO-1, major components of the tight junction, were increased in rat ileum after intermediate endurance swimming for 1 h a day, indicating that endurance exercise may decrease gut permeability [69]. However, other studies have reported conflicting results. For example, both the elements and the proportions of tight junction proteins differ between different running exercises. Nevertheless, the conflicting results indicate a relationship between physical exercise, gut permeability, and the tight junction. It was also affirmed that exercise reduces LPS-induced systemic inflammation. Moreover, many studies have proven that physical exercise suppresses chronic inflammatory diseases by influencing the gut microbiota. Overall, the effects of exercise type and exercise level, and the underlying mechanism, should be investigated intensively.

## Conclusions and prospects

**Fig. 1:**
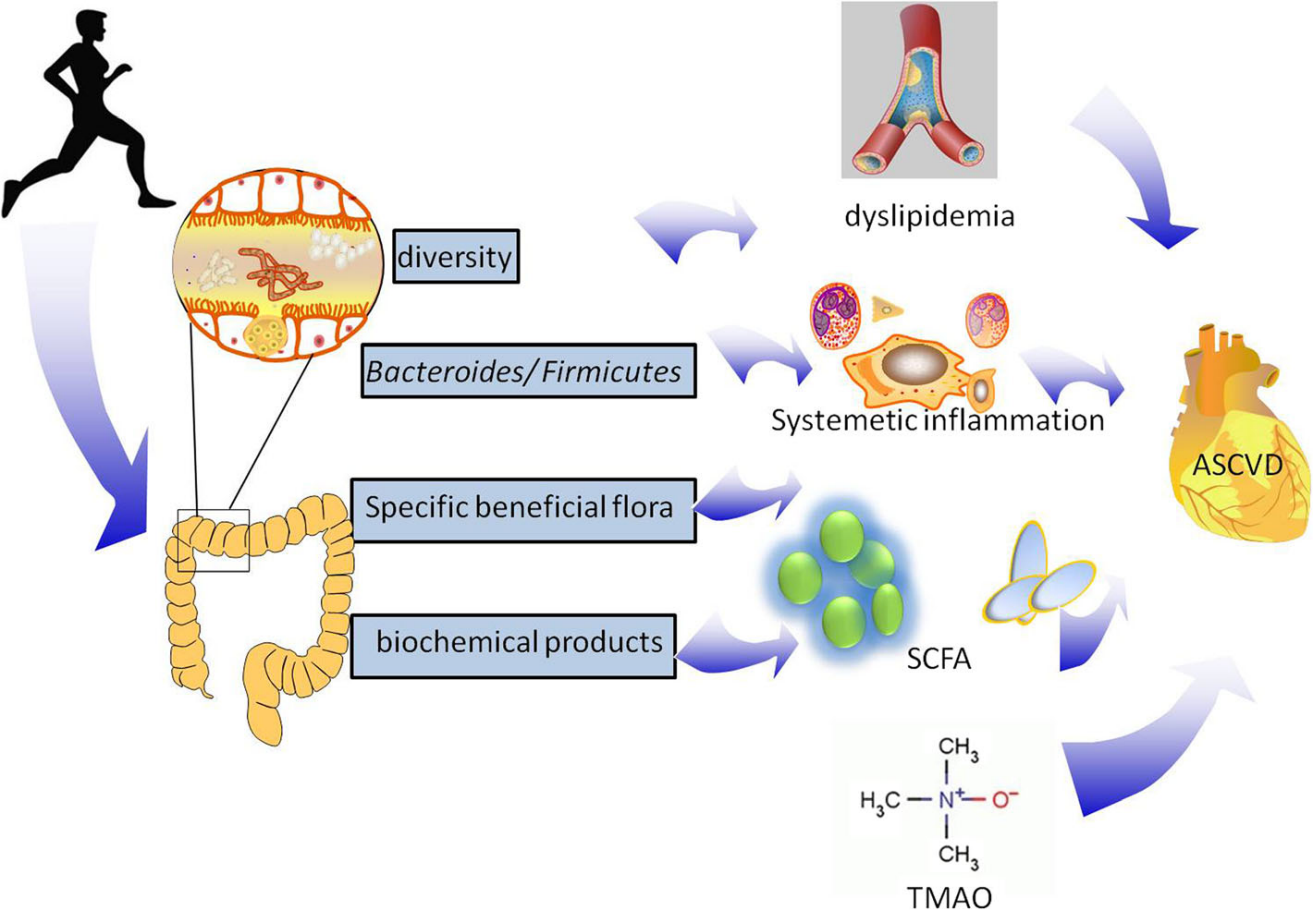
Schematic summary of effects that exercise had on ASCVD

Although no direct evidence supports the premise that physical exercise prevents ASCVD by modifying the gut microbiota and by alleviating systematic inflammation, many studies have confirmed this hypothesis. Some intestinal floras are specifically related with exercise and ASCVD, and may contain species that could ameliorate atherosclerosis by exercise Fig. 1

.

As we gain a deeper understanding of the specific relationships between physical exercise, gut microbiota and ASCVD, we expose potential therapeutic ways. However, in the field of CVDs, the gut microbiota is a newly emerging topic and has raised many questions. For example, how does exercise-induced change in the precise gut microbiota composition decrease TMA or TMAO production? What strength grade, aerobic exercise, or resistive exercise should patients undertake to achieve the best therapeutic effect? Should we undertake higher intensity exercise, low-intensity activity, or endurance training? What is the most suitable time to exercise? These questions all warrant further research.

## Abbreviations

ABCA1: ATP-binding cassette transporter A1
ASCVD: Arteriosclerotic cardiovascular disease
CETP: Cholesteryl ester transfer protein
CLDN: Claudin
CVD: Cardiovascular diseases
DMB: 3,3-dimethyl-1-butanol
dsRNA: Double-stranded RNA
HDL-C: High-density lipoprotein cholesterol
HFD: High-fat diet
ICAM-1: Intercellular adhesion molecule 1
IFN-β: Interferon-β
IP3: Inositol-1,4,5-trisphosphate
LAB: Lactic acid bacteria
LPS: Lipopolysaccharide
NF-κB: Nuclear factor-κ B
RCT: Reverse cholesterol transport
SCFA: Short chain fatty acid
SR-A1: Scavenger receptor A1
TG: Triglyceride
TLR: Toll-like receptor
TMA: Trimethylamine
TMAO: Trimethylamine *N*-oxide
VCAM-1: Vascular cell adhesion molecule 1
